# Theoretical investigations on microwave Fano resonances in 3D-printable hollow dielectric resonators

**DOI:** 10.1038/s41598-017-16501-3

**Published:** 2017-11-23

**Authors:** Eunsongyi Lee, In Cheol Seo, Hoon Yeub Jeong, Soo-Chan An, Young Chul Jun

**Affiliations:** School of Materials Science and Engineering, Ulsan National Institute of Science and Technology (UNIST), Ulsan, 44919 Republic of Korea

## Abstract

High-index dielectric structures have recently been studied intensively for Mie resonances at optical frequencies. These dielectric structures can enable extreme light manipulation, similar to that which has been achieved with plasmonic nanostructures. In the microwave region, dielectric resonators and metamaterials can be fabricated directly using 3D printing, which is advantageous for fabricating structurally complicated 3D geometries. It is therefore especially suitable for the fabrication of subwavelength structures. Here we report theoretical investigations on microwave Fano resonances in 3D-printable dielectric materials and structures. In particular, we propose and analyse 3D-printable, hollow, dielectric resonators with relatively low refractive indices, which exhibit sharp Fano resonances. We can control the interaction between bright and dark modes in a coupled dielectric particle pair by adjusting the inner-hole size, and thus we can increase the radiative Q factors further. We also find that Fano resonances in these hollow dielectric resonators are very sensitive to an index change in the surrounding medium, which could be useful for long-distance environmental sensing. New possibilities and opportunities are opening up with the rapid development of 3D-printing technologies. Our findings and the detailed investigations reported here can provide useful guidelines for future photonic devices based on 3D-printable materials and structures.

## Introduction

Metallic nanostructures can support surface plasmon resonances at optical frequencies that can be used to manipulate light at deep-subwavelength scales^1–3^. This enables the design and construction of novel nanoscale optical components and integrated circuits. Light-matter interactions also can be greatly boosted in such metallic nanostructures, due to the strong field enhancement at plasmonic resonances; this could be useful, for example, for ultra-sensitive molecular sensing. However, metallic structures suffer from intrinsic metal losses, and they often exhibit broad spectral features.

High-index dielectric structures can support multiple Mie resonances (electric and magnetic dipole and higher-order modes)^4–6^, and they can be used for similar manipulation of light. For example, semiconductor nanoparticles have been used to induce Mie resonances at visible and infrared frequencies. Such dielectric structures can be optimised to exhibit ultra-sharp spectral features, together with strong field-enhancement (*e.g*. by using Fano resonances between broad and narrow modes). In Mie resonators, various dielectric materials can be employed. Especially at microwave frequencies, there exist dielectric materials with very large refractive indices (*e.g*. ferroelectric ceramics)^7,8^, which could be useful for the fabrication of deep-subwavelength structures.

The use of dielectric materials at microwave frequencies also allow the fabrication of resonant structures *via* 3D printing, also called as ‘additive manufacturing’ because it can build an arbitrary 3D object layer-by-layer from the bottom up^9–12^. This method is advantageous for fabricating structurally complicated 3D geometries, and it is, therefore, suitable for the fabrication of microwave dielectric metamaterials and subwavelength structures. Thermoplastic or UV-curable polymers are used for extrusion-based or jet-based 3D printing, respectively, with a typical spatial resolution down to ~100 μm. This spatial resolution is suitable for the fabrication of deep-subwavelength-scale devices for the microwave region, where wavelengths are in centimetre scales (*e.g*. 10 GHz corresponds to 3 cm in wavelength). High-index microparticles (*e.g*. ferroelectric powders) can also be mixed with these polymers to increase the refractive indices of printed materials, and the composite materials can be 3D-printed into complex geometries.

**Figure 1:**
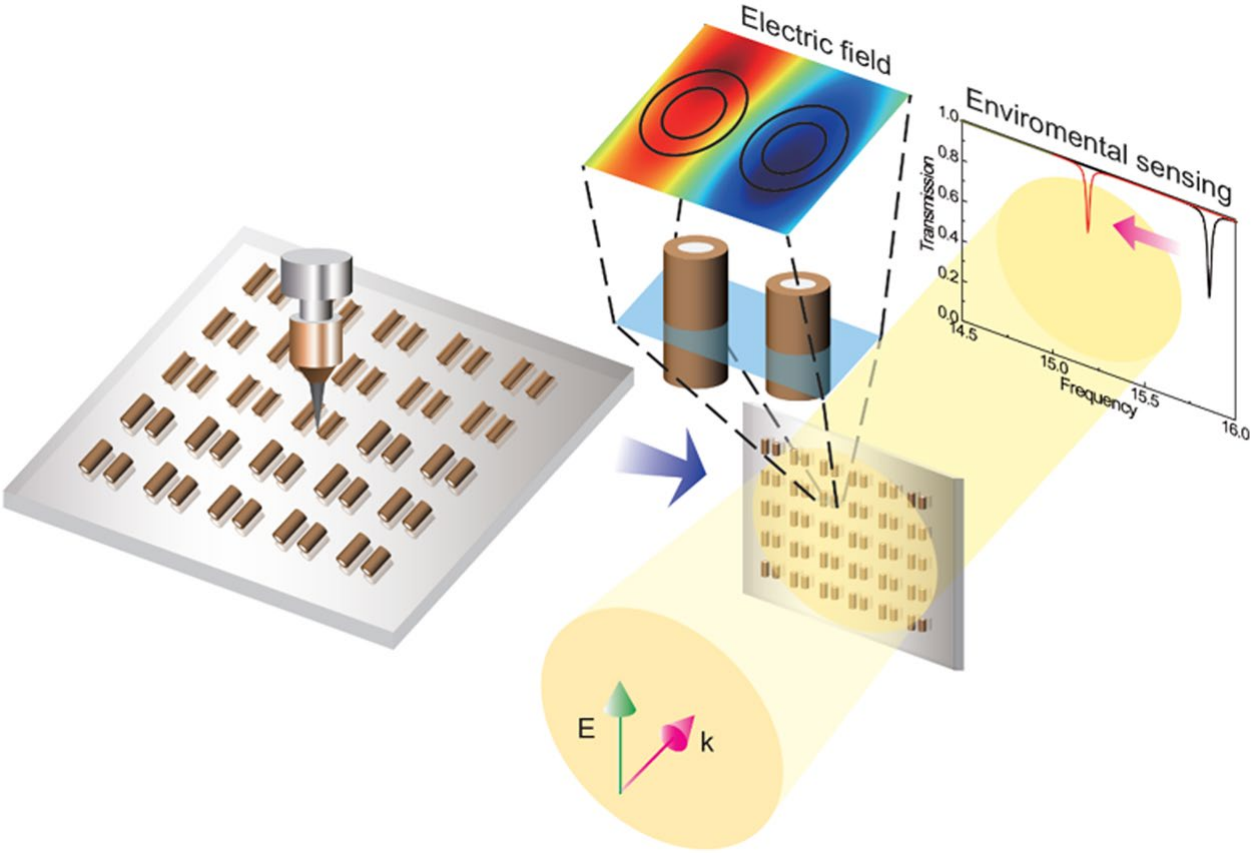
Schematic of 3D-printable dielectric resonators and their use for environmental sensing. These hollow structures could be rather inconvenient to fabricate with other manufacturing methods, but they can be readily fabricated using 3D printers. These hollow structures are very sensitive to index changes in the surrounding medium, which could be useful for long-distance environmental monitoring via microwave detection.

In this study, we have performed theoretical analyses of microwave Fano resonances based on 3D-printable dielectric materials and structures. We first investigate Mie resonances in cylindrical particles. In particular, we investigate the effects of optical losses on Mie resonances when we use high-index printable materials that employ ferroelectric powders in a thermoplastic matrix. We find that, when the material indices are increased, multiple higher-order Mie resonances appear clearly, but damping due to material losses also increases, and the Mie resonances are broadened. Alternatively, we suggest the use of 3D-printable, hollow, dielectric resonators with relatively low refractive indices (Fig. 1), which exhibit sharp Fano resonances. Such low-index materials do not suffer from serious damping due to material losses. By adjusting the inner-hole size, we can control the optical coupling between bright and dark modes in a coupled, hollow dielectric pair, and we can further increase the radiative quality (Q) factors of the Fano resonances. Such hollow structures can be rather inconvenient to fabricate using conventional manufacturing methods, but they can be produced readily by 3D-printing using typical thermoplastic materials. We also find that Fano resonances in these hollow structures are very sensitive to index changes in the surrounding medium. We observe that the sensitivity increases gradually as the inner hole gets larger. Considering both the high sensitivity and the sharpness of the resonances, we conclude that hollow dielectric resonators can be useful for long-distance environmental monitoring *via* microwave detection.

We also discuss possible future directions for 3D-printable microwave structures using multi-material 3D printing. With the rapid development of 3D-printing technologies, new possibilities and opportunities are opening up in this field. We believe that our findings and theoretical analyses can provide useful guidelines for future photonic devices based on 3D-printable materials and structures.

## Results and Discussion

### Microwave Mie resonances in a cylindrical dielectric particle

**Figure 2:**
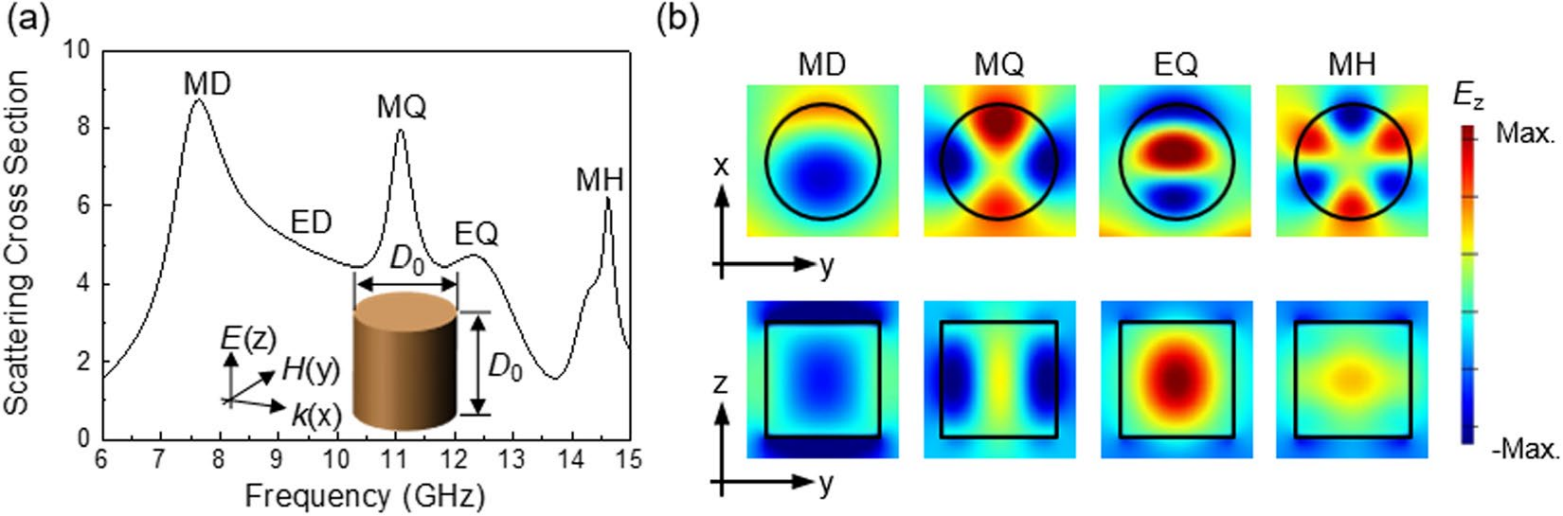
(**a**) Extinction cross section spectrum for a *lossless* single cylindrical particle with the refractive index of 2.95 and diameter (*D*_0_) of 11.4 mm. The inset shows the schematic of a cylindrical dielectric particle with incident light propagating along the x axis. The diameter and height of the particle are identical as *D*_0_. Various Mie resonance modes (electric and magnetic dipole modes and their higher-order modes) are present; MD (magnetic dipole), ED (electric dipole), MQ (magnetic quadrupole), EQ (electric quadrupole), and MH (magnetic hexapole). (**b**) Top (bottom) panels represent the *E*_z_ component of each Mie resonance mode on the xy (yz) plane. Higher order modes generally exhibit sharper resonance peaks in the spectrum, and they have more nodes (i.e., zero field lines) in the field distribution.

We first consider dielectric Mie-resonance modes supported by a single cylindrical particle. To see the various Mie resonance modes clearly, we first consider a lossless cylinder, and we subsequently consider the effects of optical losses on the Mie resonances. Figure 2(a) shows the extinction cross section obtained from the finite-difference time-domain (FDTD) simulation for a lossless cylinder with refractive index 2.95 and diameter *D*_0_ = 11.4 mm. Incident light propagates along the x axis and is polarised along the z axis, as shown in the inset. The spectrum of the extinction cross section shows various Mie-resonance modes: a magnetic dipole (MD) at 7.63 GHz, electric dipole (ED) at ~9.2 GHz, magnetic quadrupole (MQ) at 11.1 GHz, electric quadrupole (EQ) at 12.3 GHz and magnetic hexapole (MH) at 14.6 GHz^13^. The electric field (*E*_z_) distributions in the xy and yz planes are also shown for each mode in the upper and lower panels in Fig. 2(b). Both the electric and magnetic Mie resonances occur in a single cylindrical particle. Higher-order modes generally have sharper resonance peaks in the spectrum, and they have more nodes (*i.e*. zero-field lines) in the field distributions. A spherical particle supports similar electric and magnetic Mie resonances (Supplementary Fig. S1).

### Effect of optical losses on Mie resonances in high-index dielectric particles

**Figure 3:**
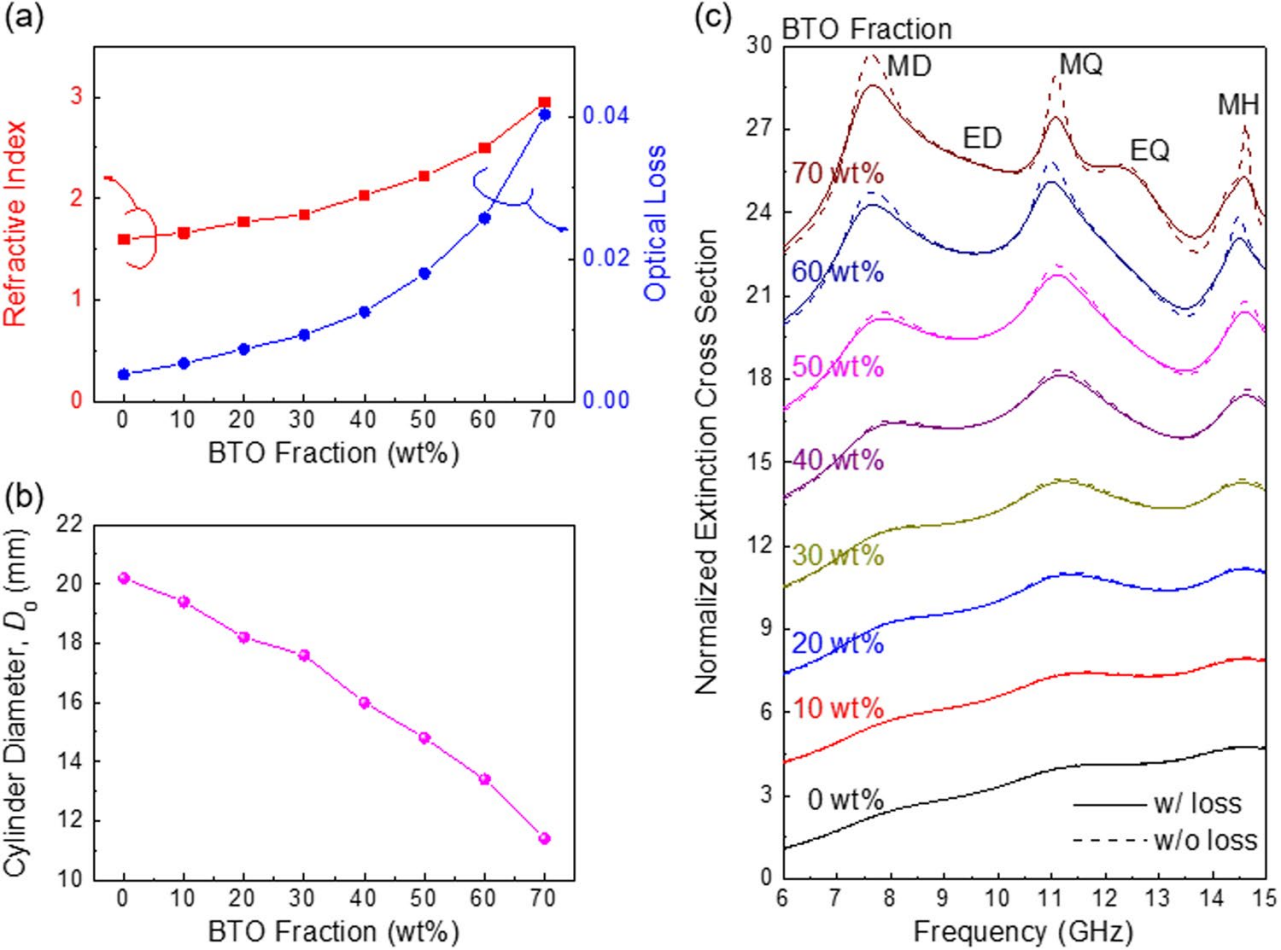
(**a**) The refractive index (*n*) and optical loss (*k*) of the BTO/ABS composite for different BTO fractions. As the BTO fraction increases, the refractive index increases gradually, but the optical loss also increases together. (**b**) The diameter of the cylindrical particle was reduced for higher BTO fractions so that we have resonances in the almost same frequency region. (**c**) BTO fraction-dependent extinction cross section for the cylindrical particles. Lossy (solid line) and lossless (dotted line) cases are shown together to evaluate the effect of optical losses.

Next we consider more realistic cases that include material losses. We use the refractive indices for a thermoplastic polymer (acrylonitrile butadiene styrene, ABS) mixed with a high-index ferroelectric powder (BaTiO_3_, BTO) that has been discussed in the literature^14^. ABS is a thermoplastic material widely used in fused-deposition-model (FDM) 3D printers. In these printers, a thermoplastic filament (with a typical diameter of 1.75 mm) is melted at high temperature (~230 °C for ABS) and extruded through a nozzle, and it is then cooled and solidified again. Thin layers are stacked, layer-by-layer, to form an arbitrary 3D object. Figure 3(a) shows the real (red line) and imaginary (blue line) parts of the complex refractive indices ($\tilde{n}=n+ik$) for different BTO *weight* fractions. We considered BTO/ABS composite filaments with eight different BTO fractions, ranging from 0 to 70 wt% in 10% increments. (Beyond 70 wt%, the filament becomes too brittle to be readily printed). As the BTO fraction increases, the refractive index increases gradually, but the optical loss also increases. The refractive index of the composite material increases to 2.95 at 70 wt%, which is the index value used in Fig. 2. Other ferroelectric microparticles can be used to increase the indices further.

To compare Mie resonances in cylindrical particles having different BTO fractions, we reduced the diameters of the cylinders with higher BTO fractions to keep the resonances within almost the same frequency range. Figure 3(b) shows the cylinder diameters we used for the different BTO fractions and Fig. 3(c) shows the spectra of the extinction cross sections, normalised by the cross-sectional area of the cylinder. We compare the realistic, lossy cases (solid lines) with the lossless cases (dotted lines) for 0 to 70 wt% BTO. We find that, as the BTO fraction increases, the higher-order Mie resonances appear clearly, due to stronger field-confinement in the high-index particles. However, damping also increases with higher BTO fractions, and the Mie resonances become weaker and broader. For low BTO fractions, the Mie-resonance peaks are very weak and are barely distinguishable. Note that both lossless and lossy particles have almost identical spectra in these low-index cases. Therefore, weak Mie resonances in samples with low BTO fractions can be attributed mainly to the low indices, not to optical losses.

Sharp resonances are often preferred for many photonic applications (*e.g*. sensing). Intrinsic material losses can potentially be reduced further by employing other ferroelectric materials. However, we instead suggest here the use of different dielectric-resonator structures, which can exhibit much sharper resonances, even with rather low refractive indices (and which are therefore free from serious damping due to material losses).

### Microwave Fano resonances in an asymmetric cylinder pair

We next consider photonic resonances in a coupled, asymmetric cylinder pair. Due to structural symmetry-breaking caused by slight differences in the sizes of the cylinders, we can obtain very sharp Fano resonances^15–24^. Initially, we consider solid, cylindrical and dielectric particles and we then investigate hollow dielectric resonators that can increase the Q factors further.

**Figure 4:**
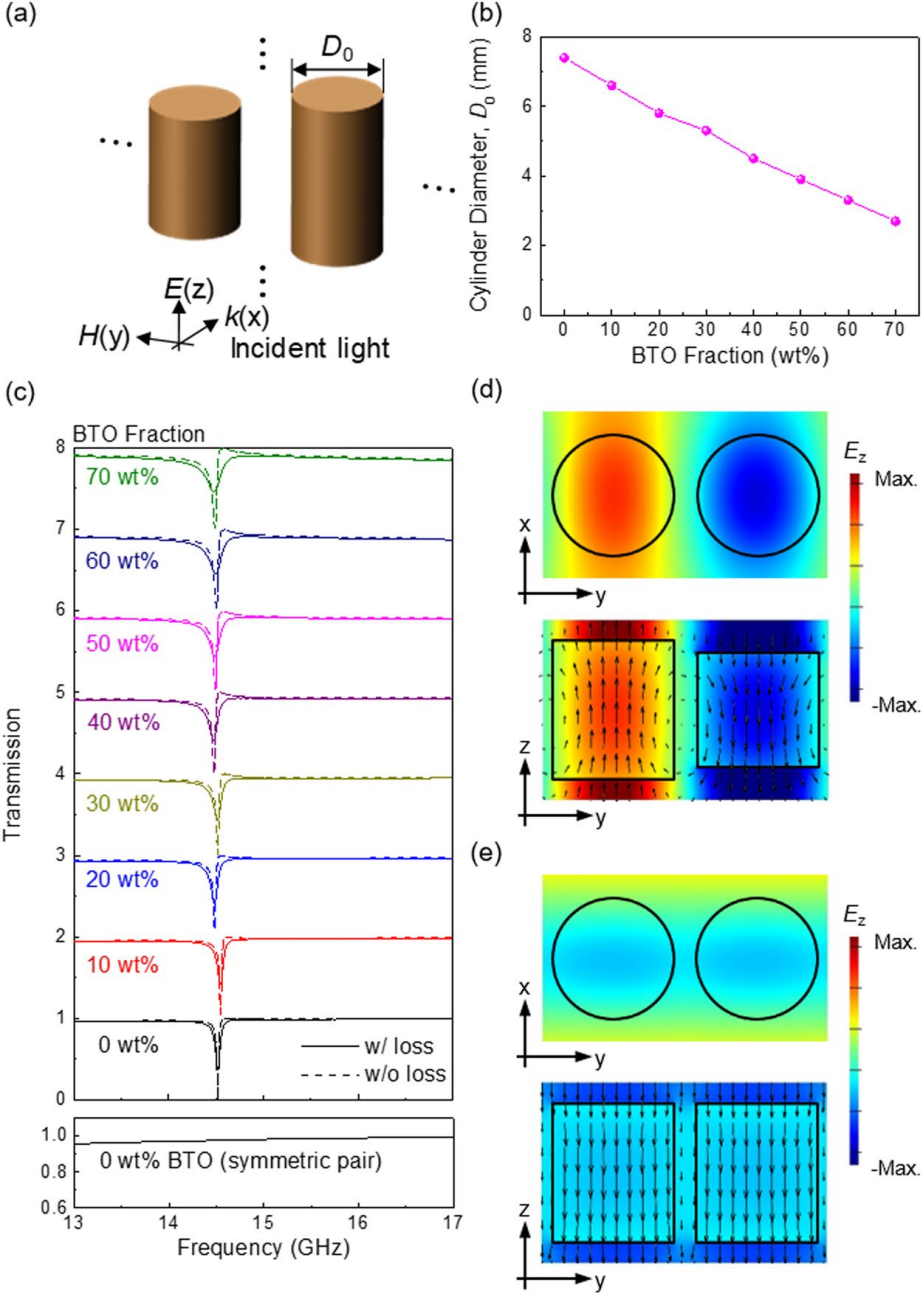
(**a**) Schematic of an asymmetric cylinder pair array. Incident light is propagating along x axis. Two cylinders are different in length (8.5 and 7 mm), while the diameter is identical as *D*_0_. The period of the array is 17.6 mm, while the distance between the two cylinders is kept as a half of the period. (**b**) The diameter of the cylindrical particle was reduced for higher BTO fractions so that we have resonances at the same frequency. (**c**) Transmission spectra for the *asymmetric* cylinder pair array for different BTO fractions. Lossy (solid line) and lossless (dotted line) cases are shown together. Sharp Fano resonances appear due to the interference between bright and dark modes. Sharp Fano resonances are obtained even for low-index materials, quite different from Mie resonances. Mie resonances did not appear clearly in such low-index structures Fig. 3(c). For comparison, the bottom panel shows the transmission spectra for the *symmetric* cylinder pair with the same lengths. (**d**) and (**e**) *E*_z_ field distribution in the *asymmetric* and *symmetric* cylinder pairs. Arrows indicate the displacement current ***D***. An anti-parallel displacement current exists in the Fano resonant, asymmetric pair only.

We consider an array of cylinder pairs with slight differences in length (8.5 and 7 mm in the z direction), as shown in Fig. 4(a). The diameters *D*_0_ of both cylinders are identical. Incident light propagates along the x axis and is polarised parallel to the cylinder axis (*i.e*. the z axis). Again, for different BTO fractions, we adjusted the diameters of the cylinders from 7.4 (for 0 wt% BTO) to 2.7 mm (for 70 wt% BTO) [see Fig. 4(b)], so that resonances occur at the same frequency (14.5 GHz). Figure 4(c) shows the transmission spectra for different BTO fractions. Sharp Fano resonances appear due to the interference between bright and dark modes. In this asymmetric, coupled, dielectric pair, symmetry-breaking plays an important role^25–28^. An anti-parallel displacement current ***D*** can be created due to a rapid change of phase around the resonance frequency. It interacts with the incident light very weakly because two opposing dipoles cancel out each other. This narrow (or dark) mode interferes with a broad (or bright) dipolar resonance in the cylindrical particles, resulting in a very sharp Fano resonance. Figure 4(d) shows the electric field distribution (*E*_*z*_) at the Fano resonance frequency, which confirms the anti-parallel field distribution between the two, asymmetric particles. The field distribution in the symmetric pair is also shown for comparison [Fig. 4(e)]. In this case, the displacement current is in the same direction in both particles, and the Fano resonance does not occur. In Fig. 4(c), we compare the Fano resonances for the lossy and lossless cylinder pairs. As the BTO fraction increases, the damping also increases, and the resonance peak broadens and the Q factors drop (see Supplementary Fig. S2 for resonance characteristics). However, for low BTO fractions, we still obtain sharp Fano resonances, which are quite different from the Mie resonances shown in Fig. 3(c). The Mie resonances did not appear clearly in such low-index structures.

### Sharp Fano resonances based on 3D-printable, hollow, dielectric pairs

**Figure 5:**
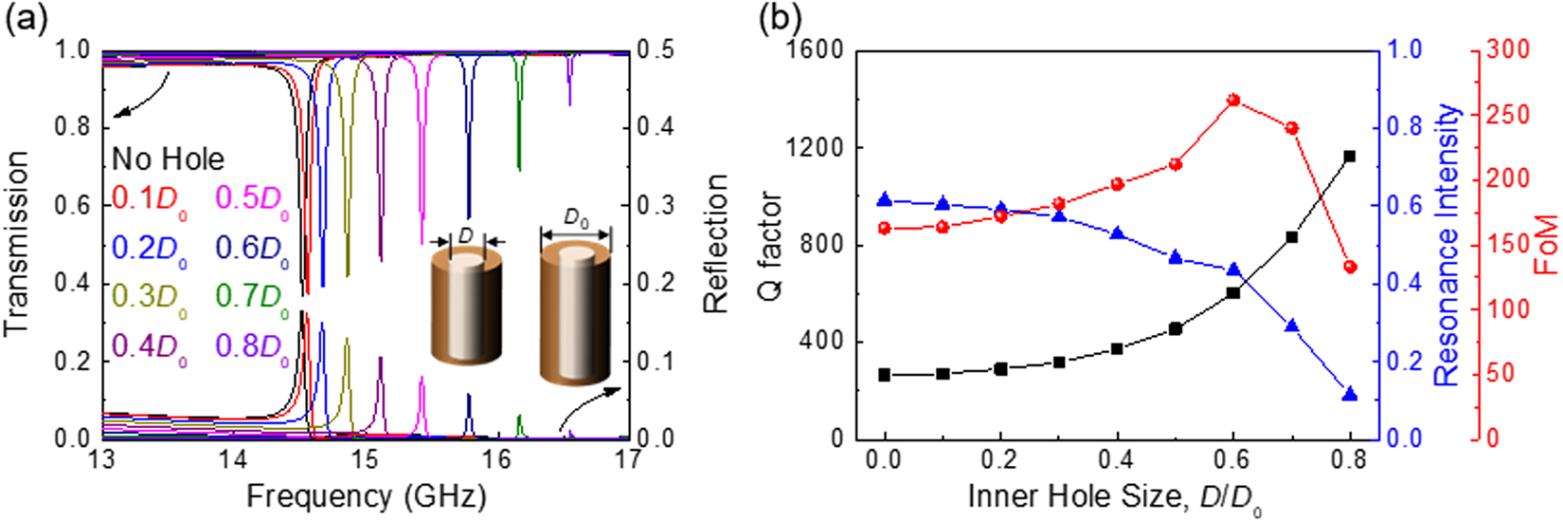
(**a**) Transmission and reflection spectra of Fano resonances in a hollow dielectric pair (see inset). The spectra obtained for different inner hole sizes with 0 wt% BTO (*n* = 1.6, *k* = 0.00376) - i.e., using a low index material with very small losses. (**b**) Resonance characteristics (Q factor, resonance intensity, and FoM) as a function of inner hole size. As the inner hole size increases, the Q factor increases but the resonance intensity decreases. As the hole size increases, the structural asymmetry between two cylinder particles decreases. The reduced structural asymmetry results in a higher radiative Q factor, together with weaker resonance strength.

We now consider hollow dielectric resonators that can increase the radiative Q factors further and which are suitable for fabrication by 3D printing. We introduce an inner hole in the 0 wt% BTO cylinder pair (*i.e*. a pair made from a low-index material, *n* = 1.6, with a very small loss, *k* = 0.00376), as shown in the inset in Fig. 5(a). The diameter of the inner hole is *D* and the height of the hole is the same as the height of the cylinder. Figure 5(a) shows the resulting Fano resonances in such hollow dielectric resonators. Both transmission and reflection spectra are shown for different inner-hole diameters. The Fano resonances are blue-shifted for larger inner holes because the effective index of the whole structure decreases. As the inner-hole size increases, the Fano resonance gets sharper (*i.e*. Q increases), but the intensity of the resonance decreases. We also observed such behaviour in ideal ‘lossless’ structures. Therefore, we think that this effect is related to the radiative optical interactions and is not simply due to the reduced volume of lossy material. In fact, the dielectric material we used in Fig. 5 has a very small loss (*k* = 0.00376).

The resonance characteristics (*i.e*. the Q factor and intensity of the Fano resonance mode) are quantitatively compared in Fig. 5(b) for different inner-hole sizes. Note that the Q factor for a Fano resonance must be determined carefully due to its asymmetric lineshape. From coupled-mode theory, the asymmetric Fano lineshape can be expressed as^17,23^

1 $$T={|{a}_{1}+j{a}_{2}+\frac{{a}_{3}}{\omega -{\omega }_{0}+j\gamma }|}^{2},$$

where *a*_1_, *a*_2_ and *a*_3_ are real-valued constants, and *ω*_0_ and *γ* are the Fano resonance frequency and the overall damping rate of the resonance, respectively. By fitting a transmission spectrum to Eq. (1), we can extract the relevant parameters, and the Q factor can then be obtained from *ω*_0_/2*γ*. We define the resonance intensity as the difference between the transmitted intensities at the peak and dip positions of the Fano lineshape. In Fig. 5(b), we plot the Q factors and the resonance intensities obtained from the transmission spectra as functions of the inner-hole size. We also define a figure of merit (FoM) for a Fano resonance as the product of the Q factor and the resonance intensity. The FoM is useful for evaluating the overall performance of a Fano resonance. Depending on specific applications, we may decide which parameter should be maximised (the Q factor, resonance intensity, or FoM).

We find that, as the inner-hole size increases, the Q factor gradually increases by up to a factor of four [black line in Fig. 5(b)], resulting in sharper Fano resonances. However, the resonance intensity drops at the same time. Consequently, the FoM reaches a maximum value at 0.6*D*_0_. Such opposite trends of the Q factor and the resonance intensity have been observed recently in other Fano resonant systems^17,18,23,24^ and were ascribed to the degree of asymmetry in the resonant structure. In our hollow structures, the structural asymmetry between the two cylinder particles decreases as the hole size increases *i.e*. the two particles effectively become more similar. When they become exactly identical (*i.e*. for a symmetric pair), the Fano resonance disappears. The reduced structural asymmetry results in a higher radiative Q factor together with weaker resonance strength. If two coupled particles are slightly different in size, the radiative Q factor of the Fano resonance can be very high, but the resonance strength can become extremely small.

### Structural-asymmetry control in hollow dielectric resonators

**Figure 6:**
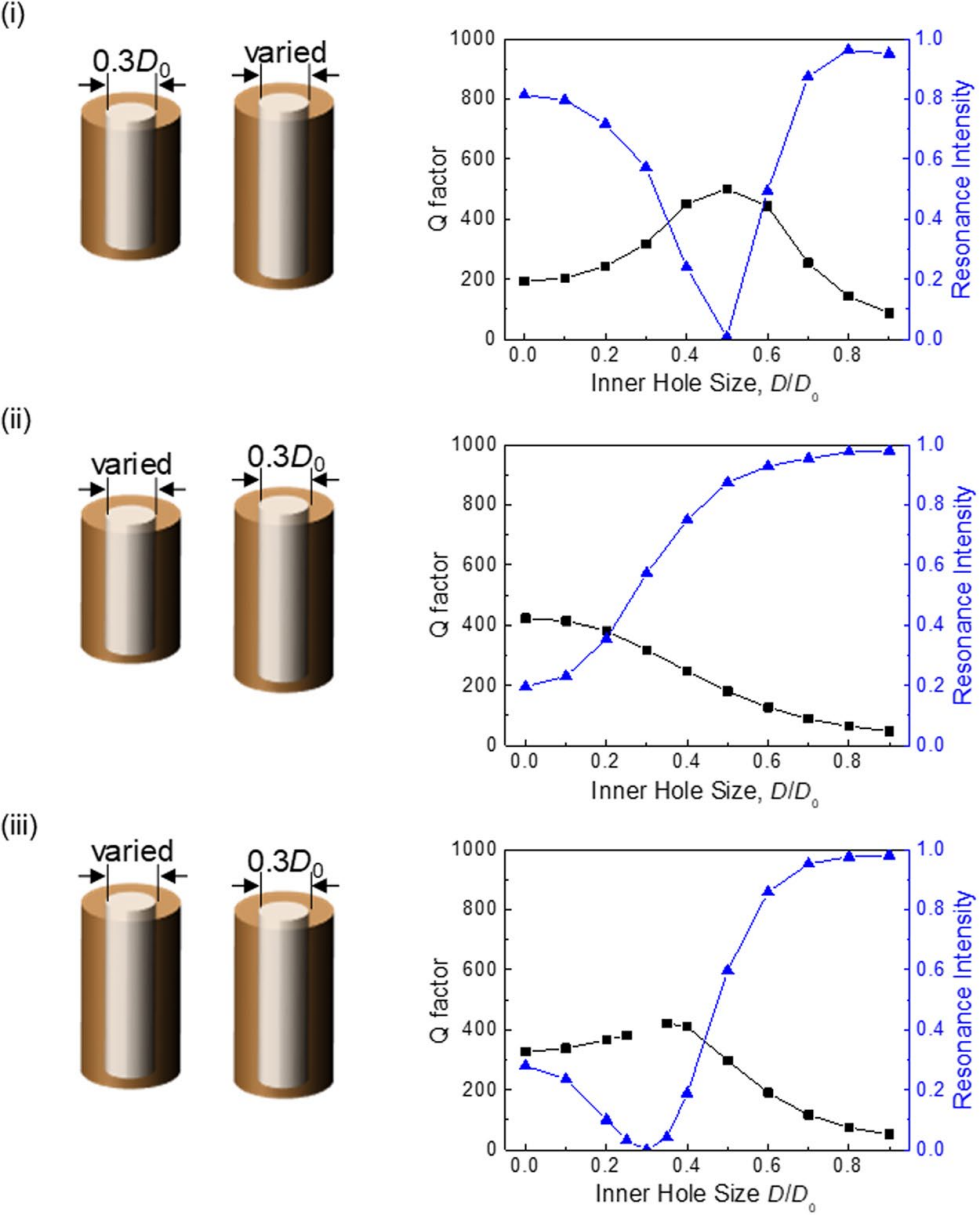
Resonance characteristics (Q factor and resonance intensity) as a function of inner hole size for three hollow dielectric structures: (i) Asymmetric cylinder pair with the hole size of the shorter cylinder fixed as 0.3*D*_0_ but the other varied, (ii) Asymmetric cylinder pair with the hole size of the longer cylinder fixed as 0.3*D*_0_ but the other varied, (iii) Symmetric cylinder pair (i.e., having the same cylinder length) with the hole size of one cylinder fixed as 0.3*D*_0_ but the other varied. All three cases can be explained based on the degree of effective structural asymmetry. By changing the inner hole size, we can adjust structural asymmetry in hollow dielectric resonators, and then we can control the Q factor and the resonance intensity of Fano resonances. In (iii), the Fano resonance *disappears* at *D* = 0.3*D*_0_, because the structure becomes exactly symmetric. So, the data point is missing at *D* = 0.3*D*_0_.

Structural asymmetry can be also controlled using slightly different geometries. In Fig. 5, the sizes of the inner holes in the two asymmetric cylinders were the same. Now we consider three more cases (Fig. 6): (i) An asymmetric cylinder pair with the hole size of the shorter cylinder fixed at 0.3*D*_0_, but with the other hole size varied, (ii) an asymmetric cylinder pair with the hole size of the longer cylinder fixed at 0.3*D*_0_, but with the other varied and (iii) a symmetric cylinder pair (*i.e*. having the same cylinder length) with the hole size of one cylinder fixed at 0.3*D*_0_, but with the other varied. We compare the Q factors and resonance intensities for these three cases and we again confirm that they have opposite trends, depending on the degree of structural asymmetry. In (i), the Q factor becomes a maximum around a hole size of 0.5*D*_0_, but the resonant intensity becomes minimum. We have the smallest asymmetry at a hole size of 0.5*D*_0_ because of the length difference between the two cylinders. In (ii), the Q factor (resonant intensity) monotonically decreases (increases) with increased hole size. When we vary the hole size in the shorter cylinder, we have the smallest structural asymmetry when there is no hole. As the hole size increases, the structural asymmetry between two cylinders gradually increases. In (iii), we have the highest Q factor near 0.3*D*_0_, but at exactly this point the Fano resonance disappears because the two cylinders become exactly identical and the dark mode no longer exists. The transmission spectra for these three cases are shown in Supplementary Fig. S3. All three cases can be explained based on the degree of effective structural asymmetry.

The opposing trends between the Q factor and the resonant intensity can be understood intuitively. The dark mode is caused by an anti-parallel displacement current ***D*** in the asymmetric dielectric pair. This mode interacts with the incident field very weakly because two opposing dipoles cancel out each other. This dark mode is coupled to a bright dipolar resonance in dielectric bars, resulting in a sharp Fano resonance. When two cylinders become more asymmetric (e.g. the size difference increases), the cancellation of two opposing dipoles become weaker. This reduced cancellation increases the coupling strength between dark and bright modes, and the energy transfer between them is also enhanced. Therefore, the coupled dielectric pair becomes more radiative; the Q factor decreases due to the radiative loss, but the resonance intensity increases because of the enhanced radiation. We also provide explanations on this opposite trend using the two-coupled oscillator model^29^, in which two oscillators are coupled with strength *g*. The oscillators represent a bright mode with resonance frequency *ω*_b_ (having a high damping rate *γ*_b_) and a dark mode with resonance frequency *ω*_d_ (having a low damping rate *γ*_d_). If they are driven by a harmonic external field, the equation of motion can be written as

2 $$\begin{array}{c}{\ddot{x}}_{b}+{\gamma }_{b}{\dot{x}}_{b}+{{\omega }_{b}}^{2}{x}_{b}+g{x}_{d}=f{e}^{iwt},\\ {\ddot{x}}_{d}+{\gamma }_{d}{\dot{x}}_{d}+{{\omega }_{d}}^{2}{x}_{d}+g{x}_{b}=0.\end{array}$$

where *x*_b_ and *x*_d_ are the displacements of the oscillators. Around *ω*_d_, where the asymmetric Fano lineshape exists, we can assume that *γ*_d_ ≪ *γ*_b_ ≪ *ω*_d_ and *ω*_b_. We can then define ${{\omega }_{b}}^{2}+i{\gamma }_{b}\omega -{\omega }^{2}\equiv C$ (*i.e*. we consider the left-hand side to vary slowly over a small frequency range around *ω*_d_). Then, the resonance width is given by ${\rm{\Gamma }}=\frac{{\gamma }_{b}{\omega }_{d}{g}^{2}}{|C{|}^{2}}$, and the modulation damping parameter is $b=\frac{{{\gamma }_{d}}^{2}|C{|}^{4}}{{{\gamma }_{b}}^{2}{g}^{4}}$. Therefore, the resonance width *Γ* and the damping parameter *b* are related to the coupling strength *g* in the opposite way: *Γ* ∝ *g* ^2^, b ∝ 1/*g* ^4^. The quantities Г and *b* are related to the Q factor and the resonance intensity, respectively; the Q factor is inversely proportional to the resonance width Γ. As discussed in ref.^29^ the damping parameter *b* determines the contrast of the Fano resonance peak (*i.e*. resonant intensity). When the degree of structural asymmetry increases, the coupling strength *g* between the bright and dark modes also increases. Therefore, the resonance width Г increases (*i.e*. the Q factor decreases), while the damping parameter *b* decreases (*i.e*. the resonance intensity increases). By changing the inner-hole size, we can thus adjust the structural asymmetry in the hollow dielectric resonators, enabling us to control the Q factor and the intensity of the Fano resonances.

### Hollow dielectric resonators for remote environmental sensing

We find that Fano resonances in hollow dielectric resonators are very sensitive to index changes in the surrounding environment. This may be useful for long-distance environmental sensing by monitoring the changes in transmission or reflection spectra in real time^19,30–33^. For example, because molecules can enter the inner hole directly, hollow structures could be of interest for environmental molecular sensing.

**Figure 7:**
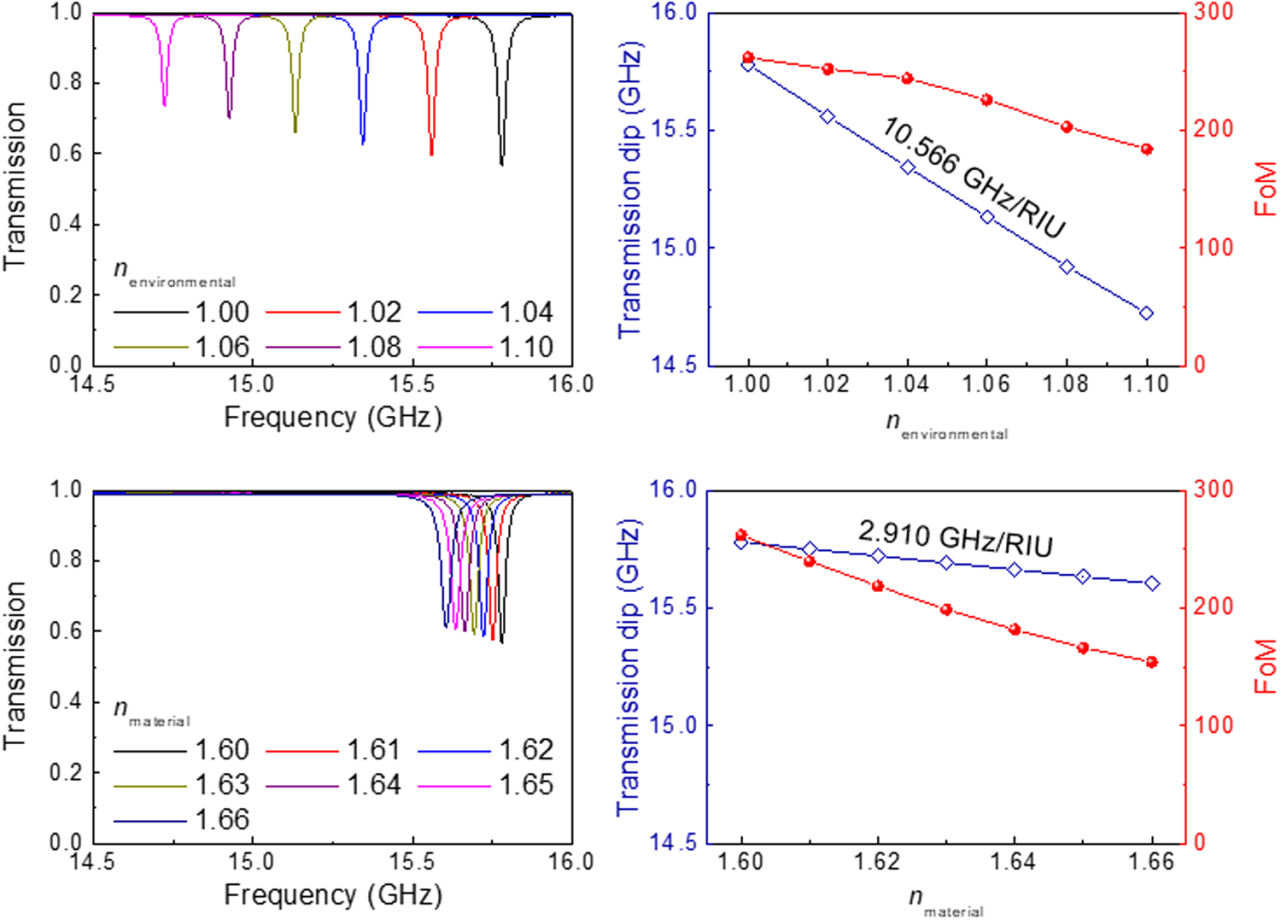
(**a**) The Fano resonance shift in the transmission spectra is shown for the cylinder pair (having the hole size of 0.6*D*_0_) with 0 wt% BTO (*n* = 1.6, *k* = 0.00376), when the environmental index, *n*_environmental_, is changed. Fano resonances in hollow dielectric resonators are found to be very sensitive to index changes in the surrounding environment. (**b**) Resonance shift and FoM for the cylinder pair array as a function of environmental index. The resonance shift is almost linear, and the sensitivity *S* is about 10.566 GHz/RIU (RIU: refractive index unit). The sensitivity gradually increases with the inner hole size, as shown in Supplementary Fig. S4. (**c**) The Fano resonance shift in the transmission spectra is for the cylinder pair (having the hole size of 0.6*D*_0_) with 0 wt% BTO, when the index of the cylinder pair, *n*_material_, is changed. The resonance shift is much smaller than that of the environmental index change in (**a**). (**d**) Resonance shift and FoM for the cylinder pair array as a function of material index. The sensitivity *S* is about 2.910 GHz/RIU.

**Figure 8:**
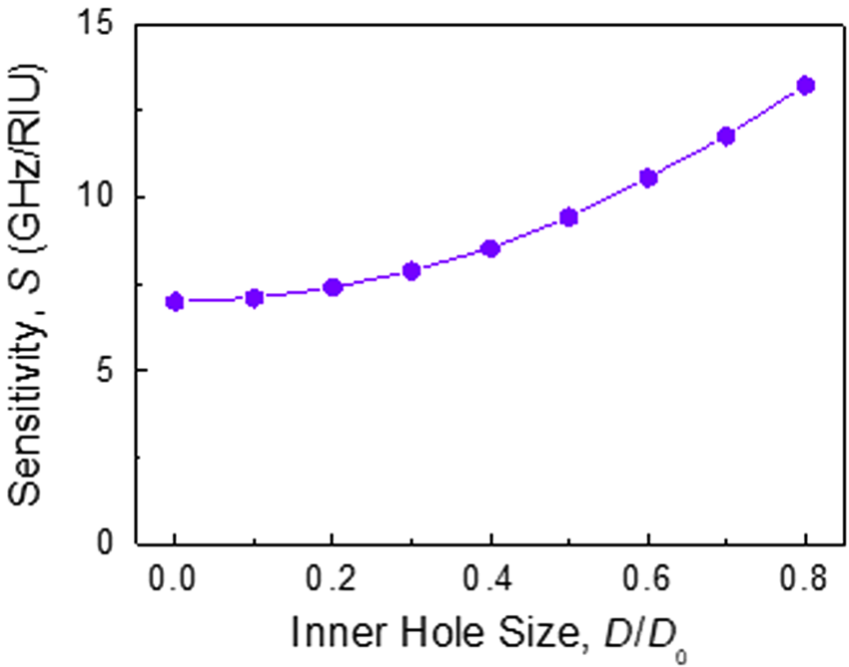
Sensitivity change as a function of the inner hole size. Here, the sensitivity *S* is defined as the shift of the Fano resonance frequency per a unit change of the refractive index (RIU: refractive index unit). The sensitivity gradually increases with the inner hole size. Considering high sensitivity values together with sharp resonance lines, we find that our hollow dielectric resonators are ideal for very sensitive environmental monitoring.

Figure 7(a) and (b) show how sensitive the Fano resonance is to environmental index changes. The hole diameter is 0.6*D*_0_, where the FoM is maximised [see Fig. 5(b)]. Figure 7(a) shows how the resonance shifts for a cylinder pair with 0 wt% BTO, when the environmental index, *n*_environmental_, changes. Even when the change is very small, *e.g*. from 1.00 (black line) to 1.02 (red line), the resonance dip in the transmission red-shifts by a significant amount. However, since the index contrast between the hollow-cylinder pair and the environment is reduced, both the resonance intensity and the FoM of the Fano resonance decrease slightly. The resonance shift is almost linear, as shown in Fig. 7(b). The sensitivity *S*, defined as the shift in the resonance frequency per unit change in the refractive index, is found to be about 10.566 GHz/RIU, where RIU denotes ‘refractive index unit.’ As shown in Fig. 8, the sensitivity increases gradually as the inner hole is made larger, from 6.983 GHz/RIU (for *D* = 0, *i.e*., for a *solid* dielectric resonator) to 13.233 GHz/RIU (for *D* = 0.8*D*_*0*_). Considering both the high sensitivity and the sharpness of the resonance lines, we conclude that our hollow dielectric resonators can be ideal for very sensitive environmental monitoring.

The refractive indices of ferroelectric ceramics are known to vary depending on the temperature, electric field, *etc* ^34,35^. Therefore, we have also considered how the Fano resonance frequency changes with the material index change. Figure 7(c) shows the resonance shift for a cylinder pair with 0 wt% BTO when the material index of the resonator, *n*_material_, increases slightly; the increased index causes a red-shift to occur. However, the amount of the shift is much smaller than that produced by the environmental index change shown in Fig. 7(a). The FoM also decreases as the material index increases. The resonance shift is again linear; *S* is about 2.910 GHz/RIU [Fig. 7(d)].

We have also studied Fano resonances in high-index (70 wt% BTO), hollow, dielectric resonators (Supplementary Fig. S4). They also show large spectral shifts with environmental index changes, but both the Q factors and the resonance intensities are lower than those in low-index resonators. Therefore, for hollow dielectric Fano resonators, it is sufficient to use low-index materials. This is in stark contrast to the Mie resonances shown in Fig. 3(c), which do not appear clearly for such low-index materials. To have sharp Mie resonances, we need high-index materials, which induce higher-order Mie resonances, but they suffer from serious damping due to material losses.

### Multi-material 3D printing for microwave resonators and metamaterials

As 3D printing technologies are developing rapidly, many new possibilities and opportunities are opening up. In particular, multi-material 3D printing can be realised using either extrusion or jet-type 3D-printers. Several different materials can be printed together using a multi-head 3D printer (*i.e*. one having several nozzles for different materials) or different materials can be mixed together before 3D printing. Multi-material 3D printing holds real promise for future photonic-device fabrication. For example, we have considered hollow dielectric resonators with an empty region inside. This inner part can be filled with another material having a higher or lower refractive index, which will enable further control of the Fano-resonance characteristics. The Mie resonances in such concentric dielectric structures could also be useful for cloaking^36^ or superscattering^37^. Using multi-material 3D printing, it is also possible to 3D-print smart materials, which can change their shapes or other properties in response to various external stimuli, such as heat, moisture, electric/magnetic fields and chemical gases, among others^38^. By including such materials in hollow dielectric resonators or in Mie resonators, we can design various microwave resonators and metamaterials which can be optimised for environmental sensing.

## Conclusion

In this study, we have performed theoretical investigations of 3D-printable, microwave Mie resonances and Fano resonances. We have found the high-order Mie resonances are very weak in low-index materials, and when high-index printing materials are employed they are significantly broadened due to material losses. However, we can obtain very sharp Fano resonances in hollow dielectric resonators with relatively low refractive indices. Such low-index materials do not suffer from serious damping due to material losses. By adjusting the inner-hole size, we can control the coupling between bright and dark modes in a coupled, hollow, dielectric pair, and we can further increase the radiative Q factors of the Fano resonances. Such hollow structures are readily fabricated using typical 3D printers. We have also found that these hollow structures are very sensitive to the index changes in the surrounding medium, which may make them useful for long-distance environmental sensing in real time. Because 3D-printing technologies are developing rapidly, we expect many more opportunities in the near future for photonic-device fabrication based on 3D-printable materials and structures.

### Data Availability

All data generated or analysed during this study are included in this published article (and its Supplementary Information files). The datasets generated during the current study are available from the corresponding author on reasonable request.

## Electronic supplementary material

Supplementary Information
