# Supplementary material for: Theoretical investigations on microwave Fano resonances in 3D-printable hollow dielectric resonators
Source: Sci Rep. 2017 Nov 23;7:16186. doi: 10.1038/s41598-017-16501-3 (PMC5701065; doi:10.1038/s41598-017-16501-3)
Supplement: Supplementary file 1 — Supplementary Information [file 41598_2017_16501_MOESM1_ESM.pdf]

Supplementary Information

Theoretical investigations on microwave Fano resonances in 3D-printable hollow dielectric resonators

Eunsongyi Lee, In Cheol Seo, Hoon Yeub Jeong, Soo-Chan An, and Young Chul Jun
School of Materials Science and Engineering, Ulsan National Institute of Science and Technology (UNIST), Ulsan 44919, Republic of Korea

Supplementary Figure S1: Extinction cross section spectra for a single spherical particle.
Supplementary Figure S2: Resonance characteristics of Fano resonances
Supplementary Figure S3: Transmission spectra of Fano resonances for three hollow dielectric pairs
Supplementary Figure S4: Fano resonance shifts due to the changes of environmental and material indices

Supplementary Figure 1

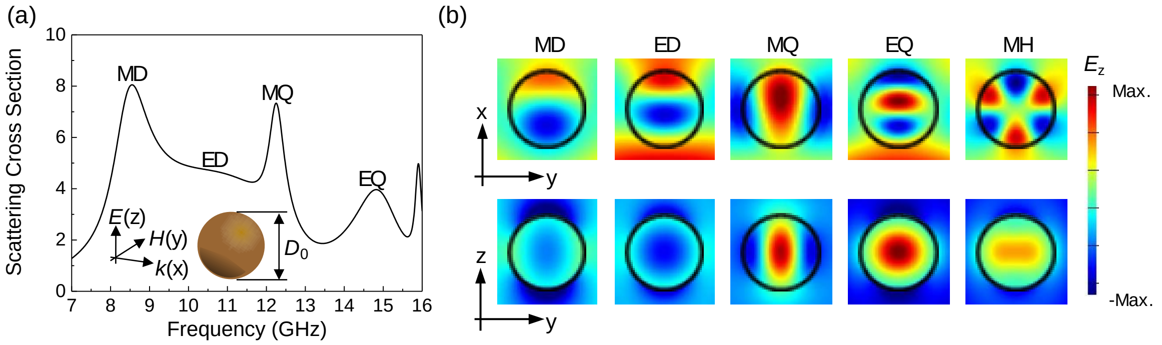

Fig. S1 Extinction cross section spectra for a single spherical particle. The inset shows the schematic of a sphere. Incident light is propagating along the x axis. The diameter of the particle is D_0. Each peak corresponds to a Mie resonance mode; MD (magnetic dipole), ED (electric dipole), MQ (magnetic quadrupole), EQ (electric quadrupole), and MH (magnetic hexapole). (b) The top (bottom) panels represent the E_z component of Mie resonance modes on the xy (yz) plane.

Supplementary Figure 2

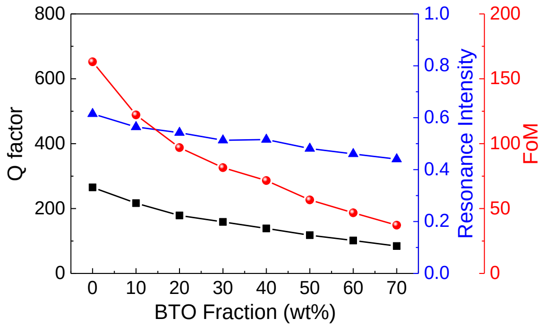

Fig. S2 Resonance characteristics as a function of BTO fraction [extracted from Fig. 4(c)]: Q factor, resonance intensity, and the figure of merit (FoM) for the cylinder pair array. As the BTO fraction increases, both Q factor and resonance intensity decreases. Therefore, the FoM drops fast with the increased BTO fraction.

Supplementary Figure 3

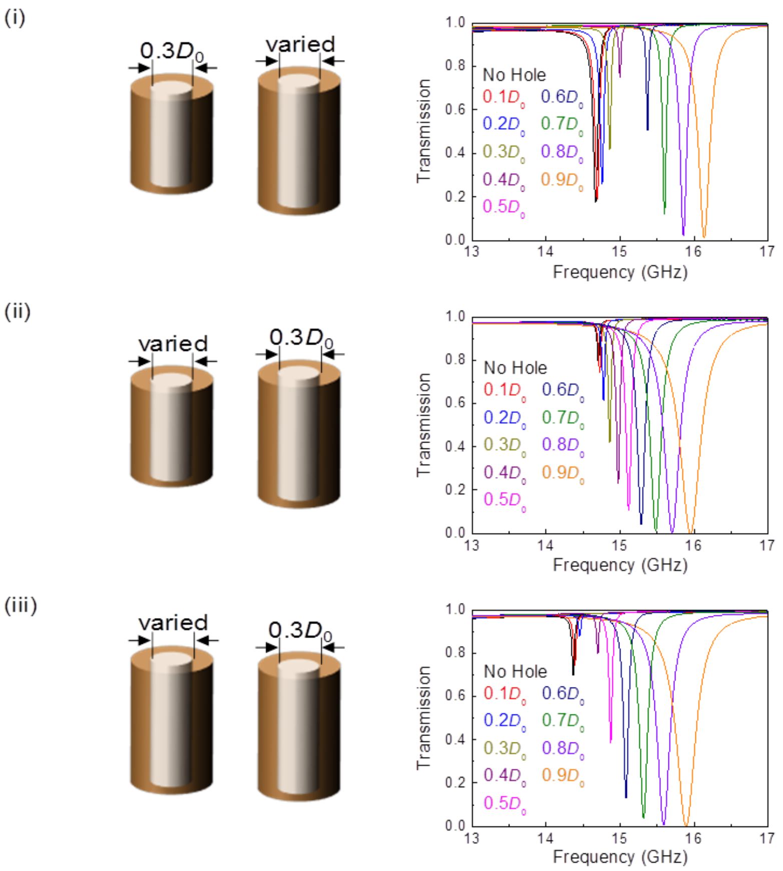

Fig. S3 (a)-(c) Transmission spectra for three different cases (corresponding to Fig. 6): (i) Asymmetric cylinder pair with the hole size of the shorter cylinder fixed as $0.3D_0$ but the other varied, (ii) Asymmetric cylinder pair with the hole size of the longer cylinder fixed as $0.3D_0$ but the other varied, (iii) Symmetric cylinder pair (i.e., having the same cylinder length) with the hole size of one cylinder fixed as $0.3D_0$ but the other varied.

Supplementary Figure 4

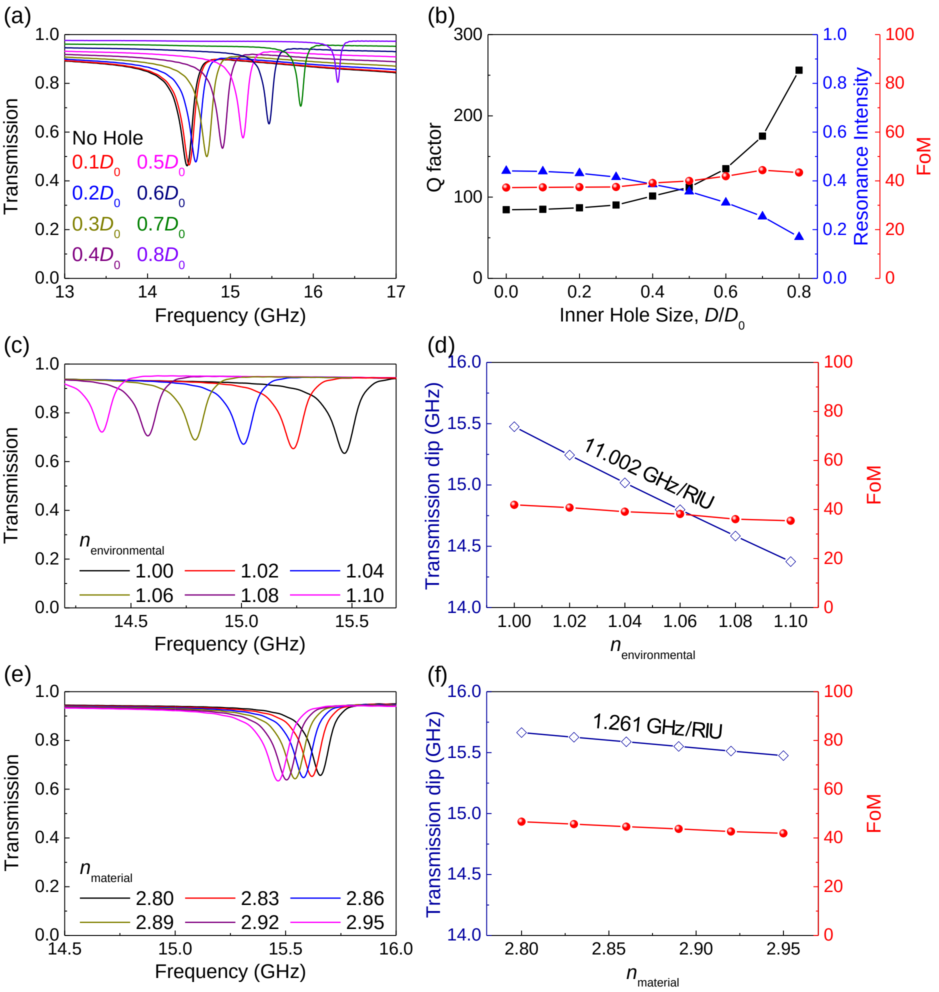

Fig. S4. **<u>Fano resonances for 70 wt% BTO fraction:</u>** (a) Transmission spectra for a hollow dielectric pair, (b) Resonance characteristics (Q factor, resonance intensity, and FoM). In (a) and (b), the resonator length is the same as that for Fig. 5 (0 wt% BTO). (c) Resonance shift for different environmental indices, (d) Sensitivity and FoM as a function of the environmental index, (e) Resonance shift for different material indices, (f) Sensitivity and FoM as a function of the material index. In (c)-(f), the resonator geometry is the same as that for Fig. 7 (0 wt% BTO). The inner hole diameter is $0.6D_0$.
